# Phthisis

**Published:** 1890-02-15

**Authors:** 


### PHTHISIS.

Dr. Thomas Harris, senior assistant physician to the Manchester Infirmary, has been lecturing on the diagnosis of the earlier stages of pulmonary phthisis (*Lancet*, November 30th). The first diagnostic points are the history of the case, including family history, and such symptoms as cough and bloody expectoration, the signification of which can scarcely be overrated. Bloody expectoration may, however, arise from several other conditions, such as spongy gums, throat ailments, nasal maladies, malingering, or it may be vicarious. Loss of flesh is also of great value as a diagnostic sign, for in the majority of cases of phthisis it is a prominent early factor. The first physical signs to be looked for are the general aspects of the chest and the remains, if present, of cicatrices of the neck, which indicate the patient has had scrofulous gland disease; while the chest may be long and narrow with small antero-posterior measurement. The posterior part of the apex of the lungs should be examined with special care, for it often gives definite results when anterior examination does not, in the way of impaired resonance. Weak vesicular murmur is very commonly heard over the apex in early phthisis, and in many cases the expiration murmur is more or less prolonged. In a second lecture in the *Lancet* of December 7th, Dr. Harris remarks that cases of early phthisis differ as regards adventitious sounds, which are sometimes present and at other times not. The most common variety is fine crepitation heard over the apex at one side. Creaks are another variety usually audible at the end of inspiration over the apex. A sibilant or sonorous rhonchus may be the only adventitious sound. If only heard over the apex it is of great significance. Often these sounds are not heard after a few deep inspirations, becoming again audible after a rest. Another adventitious sound is pleuritic friction, most frequently from dry pleurisy. Another auscultatory sign is that the sounds of the heart may be better heard over the right than the left apex in consolidation of the upper part of the right lung. But vocal resonance varies so much that it is of little value in the diagnosis of the early stage of phthisis. Temperature in early phthisis varies considerably, and is not always of assistance in diagnosis. The tubercle bacilli may be found, when, from the absence of definite signs, a positive diagnosis would have been impossible, but to find the bacilli competent and repeated observation is necessary. But, unfortunately, it is not always possible to obtain sputum from an early case of phthisis.

